# Experimental Infection of Pigs with a Traditional or a Variant Porcine Respiratory Coronavirus (PRCV) Strain and Impact on Subsequent Influenza A Infection

**DOI:** 10.3390/pathogens12081031

**Published:** 2023-08-11

**Authors:** Gaurav Rawal, Jianqiang Zhang, Patrick G. Halbur, Phillip C. Gauger, Chong Wang, Tanja Opriessnig

**Affiliations:** 1 Department of Veterinary Diagnostic and Production Animal Medicine, Iowa State University, Ames, IA 50011, USA; grawal@iastate.edu (G.R.); pghalbur@iastate.edu (P.G.H.); pcgauger@iastate.edu (P.C.G.); chwang@iastate.edu (C.W.); 2 Vaccines and Diagnostics Department, Moredun Research Institute, Penicuik, Midlothian EH26 0PZ, UK

**Keywords:** co-infection, coronaviruses in pigs, transmissible gastroenteritis virus traditional versus variant isolates, influenza A virus, pathogenicity, human disease model, porcine respiratory coronavirus

## Abstract

Porcine respiratory coronavirus (PRCV) pathogenicity in pigs has been characterized using traditional PRCV isolates; however, information is lacking on pathogenicity of currently circulating PRCV isolates. Recently, a contemporary US PRCV variant was isolated. The infection dynamics of that strain (PRCV-var) and a traditional PRCV strain (PRCV-trad) were compared. In brief, 4-week-old pigs were divided into three groups with five pigs each. The pigs were inoculated with PRCV-trad or PRCV-var, or left uninfected. Nasal swabs were collected daily, and all pigs were necropsied at day (D) 3. PRCV nasal shedding was significantly higher in PRCV-var pigs compared to PRCV-trad pigs. To investigate the impact of trad and var PRCVs on subsequent infection with influenza A virus (IAV), four additional groups of five pigs were used: PRCV-trad-IAV (PRCV-trad at D0, co-infected with IAV at D5), PRCV-var-IAV, and IAV positive and negative controls. Significantly higher mean PRCV antibody titers and a significantly higher area under the curve (AUC) for PRCV shedding were observed in PRCV-var compared to PRCV-trad-pigs at D10. There was no impact on IAV infection. In conclusion, a 2020 PRCV variant isolate was similar in pathogenicity but more transmissible compared to a traditional 1989 isolate. These findings raise concerns about virus evolution towards more highly pathogenic and transmissible strains and the need to monitor such viruses.

## 1. Introduction

Coronaviruses (CoVs) have been reported in many species, including pigs and humans. CoVs often have enteric or respiratory tropism. In humans, respiratory CoVs have been responsible for three major human pandemics in recent years, including from 2002 to 2004, the SARS-CoV-1 pandemic [1]; from 2012 onwards, the ongoing MERS-CoV pandemic [2]; and from 2019 onwards, the ongoing SARS-CoV-2 pandemic. It appears that the infection dynamics of the latter are on the verge of changing to an endemic stage. In addition to these fatal human CoVs, seasonal CoVs are circulating in humans [3].

Six CoVs have been identified in pigs, namely, four alpha CoVs, one beta CoV, and one delta CoV. Three alpha CoVs are associated with enteric manifestations in pigs and include transmissible gastroenteritis virus (TGEV) [4], porcine epidemic diarrhea virus (PEDV) discovered in 1971 in the UK [5], and severe acute diarrhea syndrome CoV (SADS-CoV) discovered in 2017 in China [6]. In addition, there is an alpha CoV with respiratory tropism known as porcine respiratory coronavirus (PRCV), first reported in 1984 in Belgium [7], in 1989 in the United States [8], and in 1992 in China [9]. Porcine hemagglutinating encephalomyelitis virus associated with respiratory and nervous system infection is a beta CoV in pigs [10]. Porcine deltacoronavirus, mainly associated with enteric infection, is a delta CoV in pigs [11].

PRCV is a deletion mutant of TGEV with 615 to 684 nucleotide deletions in the spike gene compared to traditional and variant TGEV viruses [12]. TGEV was first described in 1946 in North America and commonly causes enteric disease characterized by acute diarrhea, vomiting, and dehydration with high mortality in neonatal piglets [4]. TGEV has been reported worldwide and is endemic in North America. Sporadic outbreaks of severe diarrhea in piglets caused by TGEV continue to be reported in Asia and Europe, although the prevalence is low [12,13].

As its name implies, PRCV has a respiratory tract tropism and is commonly associated with mild-to-moderate respiratory disease in young pigs. PRCV is spread by contact or airborne transmission and can be found in pigs of all ages. PRCV is thought to spread rapidly, was found to travel several kilometers, and was found to be widespread in pigs in Europe [13]. The infection associated with PRCV is often mild or even sub-clinical, with nasal discharge and sneezing encountered in affected pigs [14,15]. Interestingly, after the emergence of PRCV, the impact of TGEV was greatly reduced, likely due to the partial immunity provided by PRCV infection [12]. PRCV is thought to be unable to bind to sialic acid, preventing infection of the gastroenteric tract [16,17]. The nucleotide deletion within the 5′ end of the spike (S) gene results in the loss of at least 200 amino acids within the N terminal region of the S protein, accounting for the loss of enteric tissue tropism of PRCV and altered disease presentation [18]. However, a recent study [19] suggests that the N-terminal domain of the TGEV spike protein may not be the sole enteric tropism determinant. Experimental studies demonstrated that pigs inoculated with PRCV shed the virus in nasal secretions for about 1–2 weeks [14,20]. Viral replication occurs in the ciliated respiratory epithelium, and PRCV-induced pneumonia in the lungs peaks between 8 and 10 days after infection [13]. Previous findings indicate that shedding, lung lesions, and clinical signs resolve after 10 days with an increase in a virus-neutralizing antibody titer against the virus [13,14,21].

The primary objective of this study was to compare the infection dynamics of a traditional (trad) PRCV strain isolated from 1989 [22], around the time when PRCV was first recognized, and a contemporary variant (var) strain isolated in 2020 [23], after PRCV had been circulating in pigs for more than 30 years. The impact of PRCV on a pig can be two-fold: induction of disease and/or potentiation of other respiratory pathogens. Hence, the secondary objective was to investigate the impact of PRCV on subsequent influenza A virus (IAV) infection. PRCV and IAV both target the respiratory tract. PRCV has been demonstrated to replicate in type 1 and type 2 pneumocytes and infects epithelial cells of the nostrils, trachea, bronchi, bronchioles, alveoli, and, sometimes, alveolar macrophages [13]. IAV replication is limited to epithelial cells of the upper and lower respiratory tract including the nasal mucosa, ethmoid, trachea, and lungs [24]. In pigs, co-infections are extremely common. As both PRCV and IAV have similar cell tropisms, it made sense to study possible interactions. The timing of pathogen interactions can be multifaceted and difficult to investigate. In this project, we simply tried to answer the question of whether existing PRCV-induced lesions, commonly present at D5, can enhance IAV infection. This study was intended to be an exploratory study. In order to properly investigate PRCV-IAV co-infections, future studies need to examine both pathogens at the same time: IAV first followed by PRCV, and PRCV followed by IAV.

## 2. Materials and Methods

### 2.1. Ethics Statement

The experimental study design was approved by the Iowa State University Institutional Animal Care and Use Committee (approval number IACUC-22-044) and the Institutional Biosafety Committee (approval number IBC-22-026) and included environmental enrichment of pens and independent veterinary supervision.

### 2.2. Pig Source, Animals, and Housing

The source farm located in Wisconsin, USA maintains a high-health herd that is regularly monitored and is free of PRCV, TGEV, IAV, porcine reproductive and respiratory syndrome virus (PRRSV), and *Mycoplasma hyopneumoniae* based on regular PCR and antibody assays. The farm routinely vaccinates all pigs with a commercial PCV2 vaccine at weaning, and the pigs used in this study also were PCV2 vaccinated at 3 weeks of age on the farm. Thirty-five, 4-week-old mixed breed and mixed-sex pigs were purchased and transported to the Iowa State University (ISU) Livestock Infectious Disease Isolation Facility (LIDIF). After arrival, the pigs were blocked by weight and then randomly divided into one of four similar-sized BSL-2 rooms (approximately 3.8 × 3.8 m), of which three rooms held two pens and one room held a single pen. In each pen, five pigs were placed. The pens were equipped with a nipple drinker and a self-feeder in which a commercial age-appropriate pelleted diet was provided to the pigs ad libitum. Simple toys for environmental enrichment were present in each pen. For the first week of housing, the rooms were acclimatized using heat lamps and rubber mats were provided for the pigs to lay on.

### 2.3. PRCV and IAV Status

The pigs were tested for the presence of anti-TGEV, -PRCV, and -IAV antibodies (serum) or RNA (nasal swabs) on the farm of origin at approximately 2 weeks of age and again at arrival at the ISU-LIDIF. Serum samples were submitted to the Iowa State University Veterinary Diagnostic Laboratory (ISU-VDL) serology section to confirm that all pigs were negative for antibodies against IAV, TGEV, and PRCV. Nasal swabs were processed for IAV and PRCV RNA and tested in our research laboratory. Details on the assays used are provided in Section 2.8 (PCR assays) and in Section 2.9 (serology assays).

### 2.4. Experimental Design

**Figure 1:**
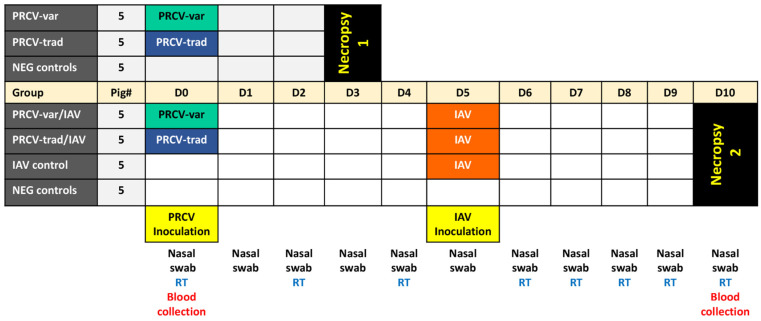
Experimental outline for investigation into porcine respiratory coronavirus (PRCV) single infection and impact of PRCV on influenza A virus (IAV) co-infection. Abbreviations: D = day post challenge. Nasal swab = collection of nasal swabs; RT = rectal temperature assessment.

Initially, we compared the early infection dynamics in pigs infected with a traditional strain isolated in 1989 [22] versus a contemporary variant PRCV strain isolated in 2020 [23]. After a 4-day acclimation period, a portion of the pigs (*n* = 20) was infected with PRCV while the remaining 15 pigs remained unchallenged. Specifically, at D0, pigs were intranasally inoculated with PRCV-trad (PRCV from 1989; *n* = 5) or PRCV-var (PRCV from 2020; *n* = 5), or left uninfected (NEG control; *n* = 5) (Figure 1).

We also investigated the possible impact of PRCV infection in pigs co-infected with IAV. In brief, pigs were experimentally infected with PRCV-trad at D0 and co-infected with IAV on D5 (PRCV-trad/IAV; *n* = 5), were infected with PRCV-var at D0 and co-infected with IAV on D5 (PRCV-var/IAV, *n* = 5), served as IAV control and infected with IAV on D5 (*n* = 5), or served as NEG control and not infected (*n* = 5) (Figure 1). The pigs were individually examined every day for signs of respiratory disease and nasal swabs were collected to determine the PRCV shedding at D0, D2, D4, D6, D7, D8, D9, and D10. Rectal temperatures were recorded every day starting at one day prior to IAV challenge. Pigs singularly infected with PRCV were necropsied at D3 while co-infected pigs were necropsied at D10 and gross and microscopic lesions were compared. These timelines for the two necropsies were chosen due to outcomes from past experiments we conducted with PRCV, where we observed that lesions with PRCV are commonly present at D3, D5, and D10, and resolved by D15 [22]. The severity of the lesions at each time point largely depends on the pig model used (gnotobiotic pigs versus SPF) and the PRCV strain used [25]. To ensure lesions were detected, we decided to necropsy a portion of the pigs at D3 and co-infect the remaining pigs with IAV with a final necropsy at D10.

### 2.5. Inoculation

All PRCV-infected pigs were inoculated with 4 mL of PRCV via the intranasal route by slowly dripping 2 mL of PRCV in each nostril. Two different virus stocks, one originally isolated in 2020 (ISU20-92330; GenBank accession number OR209254) and the other originally isolated in 1989 (AR310; GenBank accession number OR209251) were used. Both virus stocks had a titer of 10^5^ medium tissue culture infectious dose (TCID_50_) per ml and were passaged four times in the swine testicular (ST) cell line in our laboratory. The exact in vitro passage number for AR310 is unknown but our stock was likely less than 10 passages. The PRCV-2020 strain was recently isolated from lung homogenate samples obtained from Iowa [23], while the AR310 was isolated from pigs in Arkansas in 1989 [22], and frozen stocks were kept in our lab.

For the H1N1 challenge, a contemporary IAV H1N1 isolate (A/swine/Iowa/A01778877/2016/H1N1; GenBank KX960190) was used. This IAV isolate belongs to the gamma clade from 2016. The IAV isolate was passaged four times in the Madin–Darby canine kidney (MDCK) cell line. The final IAV virus titer was 10^5.5^ TCID_50_ per ml. Prior to IAV challenge, the pigs were sedated and each pig was inoculated with the final IAV stock using the intratracheal (2 mL) and the intranasal (2 mL) routes as previously described [26]. NEG control pigs were sham inoculated using negative control media intranasally using the same volume of material as that of challenged pigs.

### 2.6. Clinical Signs

All pigs in all groups were checked daily for evidence of clinical signs, and respiratory disease and cough/sneezing scores were recorded. The respiratory scores ranged from 0 to 6 (0 = normal; 1 = mild dyspnea and/or tachypnea when stressed; 2 = mild dyspnea and/or tachypnea when at rest; 3 = moderate dyspnea and/or tachypnea when stressed; 4 = moderate dyspnea and/or tachypnea when at rest; 5 = severe dyspnea and/or tachypnea when stressed; 6 = severe dyspnea and/or tachypnea when at rest), as described [27]. Similarly, cough and sneezing were scored from 0 to 2 (0 = absent; 1 = single sneeze or cough; 2 = 2 or more sneezes or coughs). The presence of sneezing and coughing were recorded during one observation period each day. Rectal temperatures were recorded in pigs one day before PRCV inoculation and at D4, D6, D7, D8, D9, and D10. All pigs were weighed at arrival (4 days prior to PRCV challenge), D3 (necropsy 1), and D10 (necropsy 2) to obtain their average daily weight gain.

### 2.7. Sample Collection

Nasal samples were collected using sterile polyester-tipped swabs (Puritan^®^, Puritan Medical Products Co., Guilford, ME, USA) placed in a 5 mL polystyrene round-bottom tube (Corning Incorporated Life Sciences, Tewksbury, MA, USA). Each tube contained 1 mL of phosphate buffered saline (GibcoTM, PBS pH 7.4 (1×), Grand Island, NY, USA), and samples were stored at −80 °C until testing. The serum samples were collected in 8.5 mL vacutainer (BD^®^, Franklin Lakes, NJ, USA) tubes. Serum tubes were centrifuged at 3000 rpm for 8 min using a Thermo scientific centrifugation machine, and serum was then poured into a 5 mL polystyrene round-bottom tube (Falcon, Corning Incorporated Life Sciences, Tewksbury, MA, USA), as described previously [28].

### 2.8. RNA Extraction and Real-Time RT-PCR

Nucleic acids were extracted from nasal swabs using the MagMAXTM Pathogen RNA/DNA kit (Thermo Fisher Scientific, Waltham, MA, USA) and a Kingfisher Flex instrument (Thermo Fisher Scientific) following the instructions of the manufacturer. For each sample, 100 uL was used for extraction, and nucleic acids were eluted into 90 uL of elution buffer as described [29]. A PRCV/TGEV/XIPC 3-plex real-time RT-PCR [30] was used to detect the shedding of the PRCV virus in nasal swabs over time in the pigs at D−4 and D0 through D10. The negative cycle threshold (Ct) cut-off value was 40. The limit of detection for PRCV was 16 copies per reaction. The PRCV standard curve values were calculated using in vitro transcribed RNA with a Y-intercept of 49.69922159, a slope of −3.509846407, and an R^2^ of 0.996932936. Similarly, a VetMAX™-Gold SIV Detection Kit (Applied Biosystems^TM^, Waltham, MA, USA) was used to detect IAV shedding [31] in nasal swabs from pigs at D5, D6, D7, D8, D9, and D10. The standard curve for IAV genomic copies was calculated based on standards obtained from the ISU-VDL. Precisely, the Y-intercept corresponded to 45.98053, the slope was −3.2976, and R^2^ was 0.995194.

### 2.9. Enzyme-Linked Immunosorbent Assay (ELISA)

A commercial influenza A virus nucleoprotein (NP) ELISA (Swine Influenza Virus Antibody Test Kit, IDEXX Laboratories, Inc., Westbrook, ME, USA) was used, and a sample to negative (S/N) ratio equal to or greater than 0.6 was considered negative while an S/N ratio less than 0.6 was considered positive. Similarly, a commercial TGEV/PRCV differential ELISA (Swinecheck^®^, Biovet^®^) was used to measure antibodies to TGEV and PRCV. A sample was considered positive for TGEV antibodies if the sample inhibition was ≤55% for TGEV and ≤40% for PRCV. A sample was considered positive for PRCV if the sample inhibition was ≤40% for PRCV and <45% for TGEV. Both assays were undertaken at the ISU-VDL serology section using serum collected at arrival, D0, and D10.

### 2.10. Necropsy and Gross Lesions

Two necropsies were performed, one at D3 (15 pigs) and one at D10 (20 pigs). Prior to necropsy, the pigs were euthanized using Fatal-Plus solution containing pentobarbital sodium (Vortech Pharmaceuticals, Ltd., Dearborn, MI, USA). The lungs were examined in a blinded fashion by a single pathologist (PGH) and given a subjective score using an established scoring system that estimates the percentage of the lung surface affected by pneumonia and consolidation [27]. In addition to lungs, nasal turbinates were also examined for evidence of inflammation and atrophy, and scores were provided from 0 = normal to 3 = severe.

### 2.11. Microscopic Lung Lesion and Immunohistochemistry

Samples of the lungs (two sections from the cranial lobe and one from each of the middle, accessory, and caudal lung lobes), trachea, nasal turbinates, and tonsils were collected, fixed in 10% neutral buffered formalin, and routinely processed for histopathological examination. The microscopic sections were examined in a blind fashion and assigned a score for the severity of interstitial pneumonia (0 to 6) [26] and necrotizing bronchitis (0 to 3) [32], as previously described.

### 2.12. Immunohistochemistry

Immunohistochemistry of paraffin-embedded blocks of lung, trachea, nasal turbinates, and tonsils for detection of PRCV [33] and IAV antigen [34,35] was performed as previously described. As PRCV cross-reacts with TGEV in tissue sections, TGEV antiserum was used. In addition, an IHC method was also used for IAV on paraffin-embedded lung sections. All IHC stains were performed at the ISU-VDL. The IHC score was based on a scoring system ranging from 0 = no antigen detectable to 3 = large amounts of antigen present.

### 2.13. Statistical Analysis

Repeated measured data including respiratory disease scores, rectal temperatures, log 10 transformed virus loads in nasal swabs determined by quantitative real-time RT-PCR, ELISA antibody titers, and average daily weight gain (ADG) were analyzed using a two-way ANOVA mixed model in GraphPad Prism version 9.3.1. Non-repeated measures of necropsy and histopathology data were assessed using non-parametric ANOVA. If a nonparametric ANOVA test was significant (*p* < 0.05), then Wilcoxon tests (with the Bonferroni correction) were used to assess the differences in pairs of groups. The area under the curve (AUC) of the log10 PRCV genomic copies over time was calculated for each pig and compared between the PRCV-trad and PRCV-var pigs. A two-sample t-test was performed to test the difference in AUC with a significant alpha level of 5%. The analysis was performed using R Statistical Software (version 4.2.2). For all analyses, a *p*-value < 0.05 was considered significant.

## 3. Results

### 3.1. Clinical Signs

#### 3.1.1. Sneezing

No sneezing was observed in the NEG control pigs throughout the study. In contrast, the mean number of days when sneezing was observed was 0.8 ± 0.4 for the PRCV-infected groups. Pigs infected with PRCV sneezed from D1 to D6. The distribution of sneezing scores 1 (single sneeze) and 2 (more than one sneeze) varied across groups. For PRCV-var pigs, six pigs with a score of 1 and eleven pigs with a score of 2 were recorded. In the PRCV-trad pigs, eight pigs with a score of 1 and four pigs with a score of 2 were recorded. Sneezing was observed again after IAV challenge from D8-10. Specifically, two pigs with a score of 1 and four pigs with a score of 2 were observed in the IAV-control pigs. In the PRCV-var/IAV group, three pigs with a score of 1 and four pigs with a score of 2 were recorded. In the PRCV-trad/IAV group, six pigs had had a score of 1 and three pigs had a score of 2. Coughing was only recorded once at D9 in an IAV control pig.

#### 3.1.2. Respiratory Scores

PRCV-infected pigs started to show mild respiratory signs by D1 (respiratory scores of 1 or 2 in two–three pigs in the groups of five pigs) and respiratory scores never went above a score of 2, except for a single PRCV-var/IAV pig that was given a score of 3 at D9.

#### 3.1.3. Average Daily Waight Gain

The ADG from D−4 to D3 was 62.7 ± 11.1 g for the NEG control pigs, and it was 16.4 ± 12.7 g for PRCV-trad and 27.2 ±12.7 g for PRCV-var with no significant differences (*p* = 0.058). The ADG from D0 to D10 was 91.9 ± 10.2 g for the PRCV-var/IAV group, 115.1 ± 16.7 g for the PRCV-trad/IAV group, 117.6 ± 21.8 g for the IAV control group, and 149.0 ± 14.2 g for the NEG control pigs. Overall, the groups had no significant difference in average daily weight gain.

#### 3.1.4. Fever

**Figure 2:**
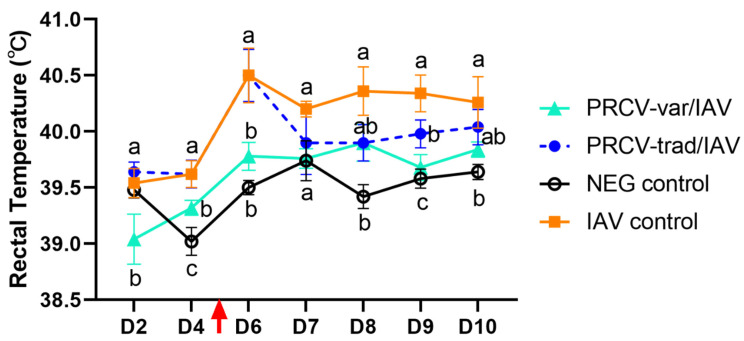
Rectal temperatures in the different groups over time. The red arrow indicates the time of the IAV challenge. Different letters (a–c) among groups on a single day indicate significant differences.

Increased rectal temperature was observed in all IAV challenged pigs at D6 but not in the NEG control pigs. The IAV control group had significantly higher rectal temperature compared to PRCV-trad/IAV on D9, whereas that of the PRCV-var group was significantly lower than that of the IAV control group at D6 and D9 (Figure 2).

### 3.2. Nasal PRCV Shedding from D0 to D10

**Figure 3:**
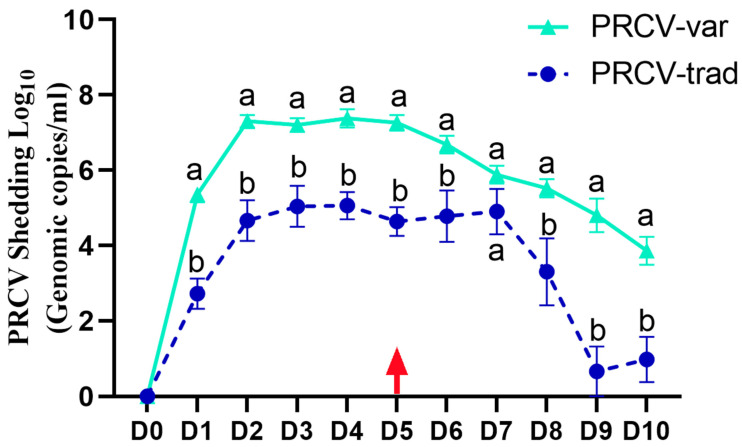
PRCV nasal shedding over time using PRCV RT-PCR. After D3, half of the PRCV-infected pigs were necropsied, and the remaining were co-infected with IAV on D5, which is indicated by the red arrow. Until D5, the differences in nasal shedding in PRCV-trad and PRCV-var groups is likely attributed to PRCV strains. Different letters (a,b) on a given day indicate significant differences between PRCV-trad and PRCV-var groups.

PRCV-var pigs had significantly (*p* < 0.05) higher RNA shedding compared to the PRCV-trad pigs over time except at D7 (*p* = 0.051) (Figure 3). The mean AUC from D0 to D10 was 59.3 ± 0.9 for the PRCV-var group and 34.9 ± 4.3 for the PRCV-trad group (*p* = 0.004). There was no impact from concurrent IAV infection.

### 3.3. IAV Nasal Shedding over Time

**Figure 4:**
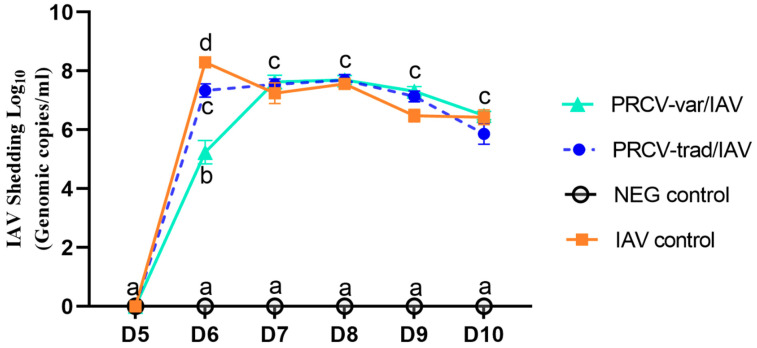
IAV nasal shedding over time using IAV RT-PCR. Data presented as mean group genomic copies. Different letters (a–d) among groups on a single day indicate significant differences.

**Figure 5:**
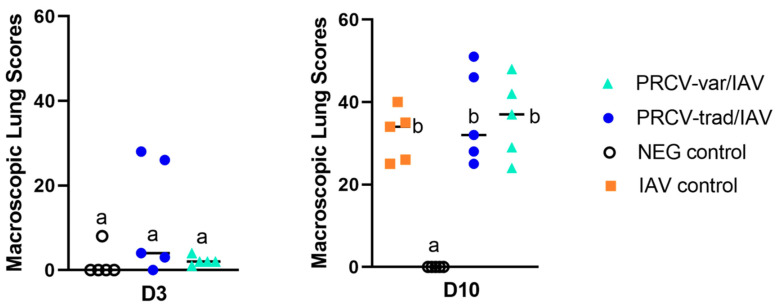
Macroscopic lung lesions in percentage of the lung surface affected by lesions (0–100%) at D3 and D10. Dissimilar letters (a,b) between groups at a necropsy day indicate significant differences.

Initially, two groups were inoculated with PRCV at D0 and these pigs were co-infected with IAV on D5. IAV shedding was uniform across the IAV positive control group and both PRCV co-infected groups (Figure 4). The IAV shedding in the IAV positive control group and both PRCV co-infected groups was significantly different compared to that of the NEG controls. At D6, one day after IAV infection, IAV control pigs had the highest number of IAV genomic copies and the PRCV-var/IAV pigs had the lowest number, which was significantly different. At later time points, there were no longer any significant differences in IAV shedding among the IAV-infected pigs, regardless of co-infection status. Negative controls remained negative over time (Figure 5).

### 3.4. Macroscopic Lesions

Macroscopic lesions are summarized in Figure 5. At D3, PRCV-infected pigs had multifocal dark red congestion areas, mainly on the dorsocaudal lung surface. While the prevalence of lesions was higher in PRCV-infected pigs compared to the NEG controls, there were no significant differences (*p* = 0.117, Wilcoxon test) in macroscopic lung lesion scores. At D10, lung lesions were most severe in co-infected pigs. There was no significant difference in macroscopic lung lesion scores between PRCV-var/IAV and PRCV-trad/IAV co-infected groups at D10.

### 3.5. Microscopic Lesions

Microscopic lesions are summarized in Table 1. Lesions were minimal in PRCV singularly infected pigs and not significantly different among groups. PRCV antigen was observed in a single PRCV-var pig. PRCV-var-challenged pigs had slightly higher amounts of inflammation and necrosis in nasal turbinates compared to PRCV-trad pigs necropsied at D3; however, this was not significant.

Interstitial pneumonia and necrotizing bronchitis associated with the presence of IAV antigen (as determined by IHC) were observed in all IAV-infected pigs at D10 (=5 days post IAV infection) without significant differences among groups (Table 1).

### 3.6. Serology

**Figure 6:**
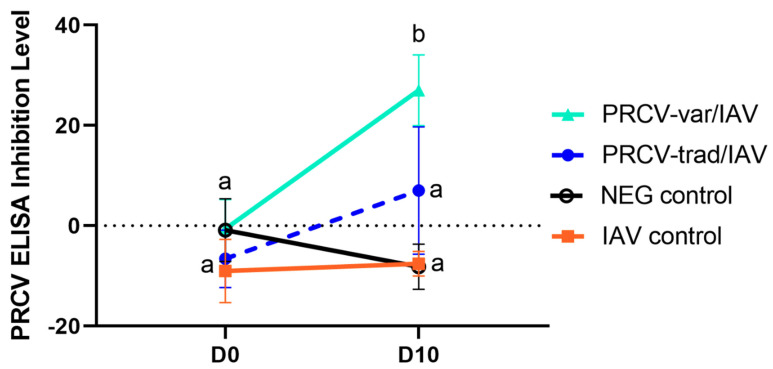
PRCV inhibition levels in serum samples collected at D0 and D10 post PRCV infection. Different letters for a group indicate a significant difference in the mean PRCV inhibition level from D0 to D10.

Based on the PRCV ELISA kit cut-offs, all samples were negative for anti-PRCV antibodies at the time of PRCV inoculation and at D10. However, as outlined in Figure 6, while the initial PRCV antibody baseline at D0 post PRCV infection was not different between the two PRCV strains (*p* < 0.05), by D10, significantly higher mean PRCV ELISA antibody titers were observed in pigs challenged with PRCV-var compared to PRCV-trad at D10. For IAV and TGEV, all pigs were seronegative on D0 and again on D10.

## 4. Discussion

Infection of pigs with historic PRCV-trad isolate or contemporary PRCV-var isolates resulted in mild respiratory disease characterized by sneezing and nasal discharge starting at D1 and lasting until D10. PRCV-var-challenged pigs had significantly higher RNA nasal shedding compared to the PRCV-trad-challenged pigs over time, except at D7. In the acute stage of PRCV infection between D1 and D6, we observed a higher number of pigs with more than one sneeze at a time (score 2) in pigs infected with PRCV-var (*n* = 11) compared to PRCV-trad (*n* = 4), which corelated with significantly higher PRCV RNA shedding in the pigs. This may indicate a possible higher virulence and/or transmissibility of the contemporary PRCV-var compared to the PRCV-trad.

In the current study, we compared a novel PRCV strain isolated during 2020 with a traditional strain, AR310, from 1989 [22]. Previously, gnotobiotic pigs infected with PRCV AR310 pigs had moderate to severe lung lesions at D10. Interestingly, no lesions were detected in nasal turbinates. Microscopic lesions were most severe at D10 and characterized by necrotizing and proliferative bronchointerstitial pneumonia with resolution of the lesions by D15 [22]. In our study using conventional pigs, we did not find any significant difference in macroscopic lung lesions between PRCV-infected groups. PRCV antigen, as demonstrated by IHC, was only detected in a single pig at D3. As this finding was observed at peak PRCV viremia, with declining PRCV titers afterwards, we did not test the D10 tissues with IHC. PRCV-var-challenged pigs had slightly higher inflammation and necrosis in nasal turbinates compared to PRCV-trad pigs necropsied at D3. Likewise, no significant difference in macroscopic lung lesion scores were observed between PRCV-var/IAV and PRCV-trad/IAV co-infected groups at D10. However, at D10, a higher prevalence of suppurative atrophic rhinitis was found in pigs infected with PRCV-var compared to all other groups.

In another study, three PRCV isolates were compared side-by-side in specific-pathogen-free (SPF) pigs inoculated with AR310, LEPP, and 1894 [25]. There was a difference in virulence among strains, with 1894 inducing only mild lesions, while AR310 and LEPP induced moderately severe lung lesions from D4 to D10 with lesion resolution by D14 in SPF pigs [25].

Researchers from the Pirbright Institute [20] inoculated pigs intranasally and intratracheally with PRCV-trad isolate AR310. No clinical signs were observed in PRCV-AR310-inoculated animals. The pigs inoculated with PRCV AR310 had fewer than 2 log_10_ genomic copies/mL of PRCV virus in nasal swabs from D1 to D5. There were no observable changes within the nasal cavity or the trachea, either grossly or microscopically [20]. In our study, slightly higher PRCV nasal shedding levels were observed, which could perhaps be explained by the pig model used or the type of PCR used.

The inflammation and necrosis in the trachea in pigs initially challenged with PRCV and at D5 co-infected with IAV were similar to those of pigs infected with IAV. This could be the result of protection provided to pigs after PRCV exposure. A similar observation was also evident with IAV vaccination and SARS-CoV2 infection using human and animal models [36,37,38,39]. Our findings may provide useful information about a model to further investigate the pathogenesis of concurrent upper respiratory tract infections in a natural host (pig) that in many ways is comparable to SARS-CoV-2 in humans.

In nasal turbinates post IAV infection, no inflammation and necrosis were seen, which is interesting. Significantly higher mean ELISA antibody titers were observed in pigs infected with PRCV-var compared to PRCV-trad, which correlates with clinical signs and PCR shedding in nasal swabs over time. PRCV and IAV both target the respiratory epithelium in pigs. Hence, prior infection of a pig with PRCV and PRCV-induced damage of the epithelial cells may result in a higher susceptibility to subsequent IAV infection. It is also possible, however, that the immune stimulation triggered by the PRCV infection could result in a faster response to IAV. Future work should investigate the impact of timing of PRCV in relation to IAV challenge, such as concurrent infection of a pig with both PRCV and IAV at the same time, in addition to investigating PRCV infection in combination with other important respiratory viruses or bacteria, including *Mycoplasma hyopneumoniae,* which also targets ciliated respiratory epithelium.

We expected that PRCV had become attenuated after circulating in pigs for over 30 years. This appears not to be the case and is good news for the pork industry; however, it is prudent to continue to monitor the evolution of PRCV and other coronaviruses in pigs using genomic sequencing tools. The PRCV-var isolated in 2020 and characterized in this study using a conventional pig model should be useful in future studies to advance knowledge about PRCV-associated molecular pathogenesis and disease ecology.

## 5. Conclusions

Infection of pigs with a traditional archived PRCV strain or a recent (2020) variant PRCV isolate resulted in mild respiratory disease characterized by sneezing, nasal discharge, and viral shedding starting at D1 and lasting at least until D10. PRCV-var-challenged pigs had significantly higher PRCV RNA shedding (*p* < 0.05) than PRCV-trad-challenged pigs except at D7 post PRCV inoculation. There was no significant difference in macroscopic and microscopic lung lesions between PRCV-infected groups. Significantly higher mean ELISA antibody titers were observed in pigs infected with PRCV-var compared to PRCV-trad, which correlated with genomic PRCV copies found in nasal swabs. IAV shedding was uniform across all inoculated groups and all pigs had similar clinical disease, indicating no impact of PRCV infection on IAV.

## Figures and Tables

**Table 1:** Microscopic respiratory lesions presented as incidence (mean group severity). Microscopic lung lesions were in the ranges of 0–6 (interstitial pneumonia) or 0–3 for necrotizing bronchiolitis, trachea necrosis, or amount of PRCV or IAV antigen straining. Differences among groups were calculated for each necropsy and are indicated by different superscripts (^A,B^).

Necropsy	Group	Lungs				Tracheal Necrosis
		Interstitial Pneumonia	Necrotizing Bronchiolitis	PRCV Antigen *	IAV Antigen *	
D3	PRCV-var	1/5 (0.2 ± 0.2) ^A^	0/5 (0.0 ± 0.0) ^A^	1/5 (0.4 ± 0.4) ^A^	Not done	1/5 (0.3 ± 0.3) ^A^
	PRCV-trad	0/5 (0.0 ± 0.0) ^A^	0/5 (0.0 ± 0.0) ^A^	0/5 (0.2 ± 0.2) ^A^	Not done	1/5 (0.2 ± 0.2) ^A^
	NEG control	1/5 (0.6 ± 0.6) ^A^	0/5 (0.0 ± 0.0) ^A^	0/5 (0.2 ± 0.0.2) ^A^	Not done	1/5 (0.2 ± 0.2) ^A^
D10	PRCV-var/IAV	5/5 (4.4 ± 0.4) ^A^	5/5 (2.6 ± 0.2) ^A^	Not done	5/5 (3.0 ± 0.0) ^A^	5/5 (1.0 ± 0.0) ^A^
	PRCV-trad/IAV	5/5 (4.0 ± 0.3) ^A^	5/5 (2.6 ± 0.2) ^A^	Not done	5/5 (2.8 ± 0.2) ^A^	5/5 (1.0 ± 0.0) ^A^
	IAV control	5/5 (4.6 ± 0.2) ^A^	5/5 (3.0 ± 0.0) ^B^	Not done	5/5 (4.0 ± 0.0) ^A^	5/5 (1.2 ± 0.2) ^A^
	NEG control	1/5 (0.2 ± 0.2) ^B^	0/5 (0.0 ± 0.0) ^A^	Not done	0/5 (0.0 ± 0.0) ^B^	1/5 (0.2 ± 0.2) ^B^

An asterisk (*) indicates the antigen was determined by immunohistochemistry.

## Data Availability

The data presented in this study are available on request from the corresponding author.
